# Habitat Selection and Behaviour of a Reintroduced Passerine: Linking Experimental Restoration, Behaviour and Habitat Ecology

**DOI:** 10.1371/journal.pone.0054539

**Published:** 2013-01-18

**Authors:** Victoria A. Bennett, Veronica A. J. Doerr, Erik D. Doerr, Adrian D. Manning, David B. Lindenmayer, Hwan-Jin Yoon

**Affiliations:** 1 Fenner School of Environment and Society, The Australian National University, Canberra, Australian Capital Territory, Australia; 2 Ecosystem Sciences, Commonwealth Scientific and Industrial Research Organisation, Canberra, Australian Capital Territory, Australia; 3 Division of Evolution, Ecology, and Genetics, Research School of Biology, Australian National University, Canberra, Australian Capital Territory, Australia; 4 Statistical Consulting Unit, School of Mathematical Sciences, The Australian National University, Canberra, Australian Capital Territory, Australia; Utrecht University, The Netherlands

## Abstract

Habitat restoration can play an important role in recovering functioning ecosystems and improving biodiversity. Restoration may be particularly important in improving habitat prior to species reintroductions. We reintroduced seven brown treecreeper (*Climacteris picumnus*) social groups into two nature reserves in the Australian Capital Territory in south-eastern Australia. This study provided a unique opportunity to understand the interactions between restoration ecology, behavioural ecology and habitat ecology. We examined how experimental restoration treatments (addition of coarse woody debris, variations in ground vegetation cover and nest box installation) influenced the behaviour and microhabitat use of radio-tracked individuals to evaluate the success of restoration treatments. The addition of coarse woody debris benefited the brown treecreeper through increasing the probability of foraging on a log or on the ground. This demonstrated the value of using behaviour as a bio-indicator for restoration success. Based on previous research, we predicted that variations in levels of ground vegetation cover would influence behaviour and substrate use, particularly that brown treecreepers would choose sites with sparse ground cover because this allows better access to food and better vigilance for predators. However, there was little effect of this treatment, which was likely influenced by the limited overall use of the ground layer. There was also little effect of nest boxes on behaviour or substrate use. These results somewhat confound our understanding of the species based on research from extant populations. Our results also have a significant impact regarding using existing knowledge on a species to inform how it will respond to reintroduction and habitat restoration. This study also places great emphasis on the value of applying an experimental framework to ecological restoration, particularly when reintroductions produce unexpected outcomes.

## Introduction

Habitat destruction and degradation are major causes of biodiversity loss worldwide [1], [2]. The ability of remaining habitat to support functioning populations of native species may be diminished because of the condition of the habitat. Therefore, restoration can play an important role in regaining functioning ecosystems and biodiversity [3], [4].

Species reintroductions will become an increasingly important part of ecosystem restoration, particularly where the poor dispersal capabilities of a species prevents natural re-colonisation. However, reintroductions must also work in concert with habitat restoration. Habitat restoration may be a necessary prerequisite to species reintroduction, especially for degraded habitats [5]. Restoration activities may aid in improving the habitat suitability of the release site, which is a vital factor influencing the success or failure of a translocation [6], [7], [8].

Unfortunately, knowledge of how to restore habitat is hampered by the under-utilization of an experimental framework within restoration projects [9]. Furthermore, the effectiveness of restoration efforts is typically assessed by analysing the resulting species composition and richness within the habitat [10], [11]. Yet, such techniques do not establish a functional connection between species presence and the experimental treatments, or necessarily reflect the quality of the restored habitat and its effect on species survival and reproduction [12], [13]. An improved understanding of how individual animals utilise habitat and the importance of particular resources can be obtained through examination of how they behave in restored habitat [11], [14]. Behavioural patterns can provide valuable information for conservation biology [15] by revealing information on food availability [14], foraging preferences in different habitats [16], and factors influencing reproductive success [17]. In restored environments, documentation of patterns of behaviour and microhabitat use can verify whether or not the effects of habitat features are as predicted for a particular species based on prior ecological information in intact environments. Thus, species behaviour can identify variation in habitat quality, act as a bio-indicator for the success or failure of restoration treatments [18], and hence inform land management and further ecological understanding.

Mulligans Flat and Goorooyarroo Nature Reserves are temperate woodlands in south-eastern Australia that are currently being restored through a large-scale experiment [19]. Experimental manipulations throughout the reserves include the addition of 2,000 tonnes of coarse woody debris, variation in ground vegetation cover (partly through management of kangaroo grazing) and the installation of nest boxes. We reintroduced the brown treecreeper, *Climacteris picumnus*, a bark and ground-foraging and hollow-nesting passerine, into these reserves as a part of this ecosystem restoration experiment. Seven brown treecreeper social groups, comprised of 43 individuals, were released in November-December 2009 [20]. These social groups were sourced from two wild populations in the Murrumbidgee region of New South Wales.

The brown treecreeper recently disappeared from the reintroduction site possibly due to the effects of habitat degradation and fragmentation [21], [22], [23]. However, the experimental manipulations within the reserves were specifically designed to ameliorate any effects of habitat degradation for this and other ground-foraging birds, which are declining throughout their range [23], [24]. In particular, coarse woody debris provides refuges for treecreepers from predators [25], [26], as the species flees to nearby hollows in trees or logs when under threat from air-borne predatory or aggressive species [27]. The species may act less cautiously (i.e. forage more on the ground) when near these structures, since an individual’s distance from a refuge is likely to influence its perceived predation risk [28], [29], [30]. Further, added coarse woody debris has increased invertebrates within the reserves [31], and provides suitable foraging substrates for the brown treecreeper [32], [33].

Variations in the level of ground vegetation cover (enhanced by areas excluding kangaroo grazing) may influence the accessibility of food [34], [35] and the perceived predation risk of individuals foraging in the ground-layer [36]. A relatively low level of ground vegetation cover has been associated with increased reproductive success for the brown treecreeper because it may improve foraging efficiency and facilitate the detection of, and the escape from, predators [37]. Finally, installed nest boxes may provide additional escape hollows when birds are under threat from predation (along with providing opportunities for nesting). Therefore, the brown treecreeper is an appropriate focal species for testing the effectiveness of restoration treatments [18].

In the reintroduction program we report here, we analysed the behaviour and micro-habitat use of reintroduced brown treecreepers to examine how well the species responded to habitat restoration and hence also the effectiveness (or success) of the restoration actions. We hypothesised that the addition of coarse woody debris, the maintenance of relatively low levels of ground vegetation cover, and the installation of nest boxes would improve the habitat for this species, which would be reflected in the use of particular behaviours and substrates in these different treatment areas. Specifically, we predicted that:

1. Increased levels of ground vegetation would decrease the probability of individuals using the ground layer, particularly for foraging.
2. Increased levels of ground vegetation would reduce the ability to detect predators and thus decrease the probability that individuals would display vulnerable behaviours (resting and preening), particularly when on the ground and on logs.
3. Increased levels of ground vegetation would reduce the ability to detect predators and thus increase the probability that individuals would display vigilance, particularly when on the ground and on logs.
4. The addition of coarse woody debris would increase the probability that individuals would forage on the ground and on logs.
5. The addition of coarse woody debris would provide more refuges from predators and aggressive species and thus increase the probability that individuals would display vulnerable behaviours (resting and preening) and decrease vigilance.
6. The installation of nest boxes would provide more refuges from predators and aggressive species and thus increase the probability that individuals would display vulnerable behaviours (resting and preening) and decrease vigilance.

Testing these predictions through an experimental reintroduction successfully integrated three sub-fields of ecology that are typically addressed separately: restoration ecology, habitat ecology and behavioural ecology. This study will provide a greater understanding of 1) the effectiveness (or success) of ecosystem restoration, 2) the biology and behaviour of the brown treecreeper, and 3) the reintroduction process, including the importance of habitat suitability and the utilization of existing knowledge on species populations to inform species reintroductions.

## Materials and Methods

### Ethics Statement

This study was conducted in strict accordance with animal ethics approval obtained through The Australian National University Animal Experimentation Ethics Committee, which specifically approved this study (C.RE.55.08). All reasonable actions were taken to minimise the impact on the welfare of the animals involved, including utilising appropriate methods for the capture, transport and monitoring of reintroduced brown treecreepers.

The project was conducted under a Scientific Licence (licence number S12906) and an Export Licence (licence number IE095650) both issued from the New South Wales Office of Environment and Heritage. The study was also issued a Licence to Import (licence number LI2008330) from the Australian Capital Territory Department of Territory and Municipal Services. Accessed land was a mixture of private property, travelling stock reserves managed by the Hume Livestock Health and Pest Authority and Nature Reserves managed by the Australian Capital Territory Department of Territory and Municipal Services. Full details of the capture, transportation and release of reintroduced brown treecreepers are provided in Bennett et al [20]. We did not sacrifice any individuals.

### Study Area

Mulligans Flat Nature Reserve and Goorooyarroo Nature Reserve are located in the Australian Capital Territory and were established in 1995 and 2004 respectively. They were previously leasehold grazing land. In total, the reserves cover 1623 ha of predominantly partially-modified lowland temperate woodland and dry forest [38]. Australia’s temperate woodlands are an extensively modified ecosystem [39], [40]. Human-induced disturbances within temperate woodlands include vegetation clearing and fragmentation, removal of coarse woody debris for firewood and fencing, livestock grazing, the loss of mature trees (an important source of nesting hollows), the invasion of exotic species, and the dominance of aggressive species such as the noisy miner, *Manorina melanocephala* [38], [39], [40], [41]. Restoring such habitats within an experimental framework is highly desirable [19], [39]. Mulligans Flat Nature Reserve has an 11.5 km mammalian predator-proof fence erected around its perimeter which excludes predators such as feral cats and the red fox (*Vulpes vulpes*), and will therefore allow reintroductions of locally extinct native mammal species in future years.

Mulligans Flat and Goorooyarroo Nature Reserves are the location of the ‘Mulligans Flat – Goorooyarroo Woodland Experiment’ [19], [38]. This experiment aims to quantify biodiversity responses to restoration treatments within temperate woodlands [19]. For that experiment, the reserves were stratified into ‘polygons’ according to vegetation type and structure. Twenty-four polygons containing woodland were selected as experimental polygons. Each experimental polygon was subject to the addition of 80 tonnes of coarse woody debris (CWD) in an attempt to reverse the negative effects of previous removal of timber over the past 150 years. The addition of CWD in each polygon was arranged within four 1 ha sites: (1) no added CWD; (2) 20 tonnes of CWD in a dispersed pattern; (3) 20 tonnes of CWD distributed to mimic a tree fall (clumped); and (4) 40 tonnes of CWD with both dispersed and clumped distributions. In addition, the intensity of grazing across the reserves, and thus the cover and biomass of ground vegetation, was manipulated through the creation of kangaroo exclusion areas [19]. Experimental manipulations within the reserves commenced in spring 2007.

### Experimental Framework

We classified each of the experimental polygons according to two additional experimental treatments: 1) high or medium ground vegetation cover; and 2) the presence or absence of nest boxes. We categorised ground vegetation cover using data on vegetation characteristics collected by McIntyre et al. [42]. We extracted data on total biomass and live plant basal area of all herbaceous plants plus sub-shrubs (<50 cm tall) for each polygon. We incorporated both basal area and biomass since both could influence ground layer quality and the manoeuvrability of brown treecreepers while ground-foraging. We created standardised scores of each of these variables (Student’s t statistics, i.e. z-scores for a population that has only been sampled and is not fully known) and summed the scores to create a single measure for ground vegetation. We then ranked the experimental polygons according to this measure to create categories of ground vegetation cover (medium and high), with the lower 50% classified as containing ‘medium’ levels of ground vegetation (average score −1.00; range −2.17 to −0.16) and the upper 50% classified as containing ‘high’ ground vegetation (average score 1.07; range −0.11 to 3.99).

Brown treecreepers also utilised areas that were outside the experimental polygons previously established. These areas were used during the extensive dispersal of individuals, but also after settlement as final home ranges [43]. For these areas, we classified non-experimental woodland areas as medium or high ground vegetation cover through comparison with experimental polygons. If a non-experimental area was dry open forest, we assigned it a ‘low’ level of ground vegetation cover, since Australian dry open forest typically contains a greater density of trees than woodland, which is associated with a lower level of ground vegetation cover [44], [45], [46].

Finally, we installed 216 species-specific nest boxes in half (12) of the experimental polygons (six in high and six in medium ground vegetation cover) distributed uniformly across the nature reserves. We clustered nest boxes (40 cm deep, 10×10 cm base, 5 cm hollow opening) on trunks of large trees (four or five per tree) to make them more apparent to the brown treecreeper, and placed them between four to eight metres above ground, which was within the normal range of nest heights. We designed the nest boxes using knowledge of the behaviour and natural nesting hollow dimensions of the brown treecreeper, as collected by Noske [26], while also aiming to reduce competition with other cavity-using species like the common starling (*Sturnus vulgaris*).

### Study Species

The brown treecreeper is a woodland dependent bird that nests and roosts in naturally-occurring tree cavities in a variety of eucalypt species [26]. The species is almost entirely insectivorous, spending between 51% and 65% of foraging time on the ground [32], [33], [47]. The species is a facultative cooperative breeder, living predominantly in gregarious social groups comprised of a breeding pair and a number of offspring that have delayed dispersal [48], [49]. Social groups of the brown treecreeper occupy territories averaging 3–6 ha in size in higher quality habitat [49], [50].

The brown treecreeper persisted in our study area until 2005 (Jenny Bounds, Canberra Ornithologists Group, personal communication), suggesting that many of the requirements for survival may still be present, particularly in comparison to the requirements for species that disappeared from the reserves many years ago. The nature reserves currently sustain other ground-foraging insectivorous species including the yellow-rumped thornbill (*Acanthiza chrysorrhoa*) and the scarlet robin (*Petroica boodang*).

### Behaviour and Micro-habitat Use

We released each of seven brown treecreeper social groups (of between four and eight individuals) in a unique polygon representing a combination of the ground vegetation and nest box experimental treatments. At release, we fitted eighteen adult brown treecreeper individuals (average weight 30.39 g, ranging from 27.50 g to 37.00 g) with radio-transmitters (Holohil Systems Model BD-2). The weight of the transmitter (0.90 g) represented 2.8% of the average bird weight. Radio-transmitters of this kind have been used extensively in brown treecreeper studies in the past [49], [51], [52].

We radio-tracked individuals daily after release from November-December 2009 until February 2010. Upon location and identification of a radio-tracked individual, we observed it for 30 seconds prior to recording an instantaneous observation of behaviour, microhabitat use and global position. We recorded observations from at least 30 metres away from individuals. If birds reacted to our presence (such as by fleeing or alarm calling) we moved away and waited 10 minutes before recording further observations. We assigned behaviour to the following categories: (1) foraging; (2) resting; (3) preening; (4) calling; (5) vigilance; or (6) other (see Information S1 for category descriptions). We defined microhabitat use as the substrate on which an individual bird was observed using the following categories: (1) bare ground; (2) leaf litter; (3) grass; (4) trunk; (5) branch; (6) log; or (7) other. We also recorded whether an individual was located within two metres of a log. We recorded the global position (UTM coordinates) for each location to determine the polygon in which an individual was located, and hence the level of ground vegetation cover, whether or not a nest box was located within the polygon, and whether or not the individual was within a 1 ha CWD site within the polygon. We located and recorded observations for each radio-tracked individual at least twice per day.

### Statistical Analyses

We conducted preliminary analyses to examine whether brown treecreeper behaviour or substrate use was influenced by the reintroduction process. We performed a two-sample binomial test for each behaviour and substrate individually. This test compared the number of observations of the target behaviour or substrate before and after the establishment of a home range, in relation to the total number of observations recorded in the respective time period. Movement prior to the establishment of a home range was taken as an “adjustment period” during which individuals may exhibit altered behaviour due to being unfamiliar with their environment or as a reaction to the reintroduction process [53], [54]. We determined the point at which a social group settled and established a home range using the methods described in Bennett et al. [43]. Data was included only for those individuals for whom we had obtained data both pre- and post-settlement. The results of these analyses indicated that there was little difference in the observations recorded pre- and post-settlement (Table 1). Therefore, for all subsequent analyses, we examined the combined dataset (pre- and post-settlement).

**Table 1:** The effect of translocation on behaviour and substrate use.

Target characteristic	% pre-settlement	% post-settlement	P value	95% CI
Foraging	50.46	55.12	0.112	−0.104, 0.011
Resting & preening	11.15	13.98	0.147	−0.066, 0.010
Vigilance	30.53	27.15	0.204	−0.018, 0.086
Branch	20.29	14.47	**0.009**	0.015, 0.102
Ground	11.88	8.94	0.100	−0.006, 0.065
Log	19.74	19.67	0.976	−0.045, 0.047
Trunk	45.34	55.61	**<0.001**	−0.160, −0.045

Results of preliminary analyses comparing the behaviour and substrate use of reintroduced brown treecreeper individuals pre- and post-settlement after reintroduction.

Our analyses examined the effects on reintroduced brown treecreepers of restoration efforts, specifically the addition of coarse woody debris and nest boxes, and ground layer management. We initially examined whether there was a broad-ranging effect of the experimental treatments on behaviour (i.e. irrespective of the substrate the behaviour was associated with). We then conducted further analyses by separately examining differences in substrate use for particular behaviours.

To test our predictions that specific behaviours should be exhibited more frequently in the various experimental treatments, we constructed Binomial Generalized Linear Models (GLM) and Binomial Generalized Linear Mixed Models (GLMM) [55], [56]. We treated three target behaviours as response variables: (1) foraging; (2) vulnerable behaviour (in the form of resting and preening); and (3) anti-predator behaviour (in the form of vigilance). For each model, we characterised the target behaviour as 1 and all other behaviours as 0. Due to the low number of observations for some substrate categories, we combined some categories to give five categories: (1) ground (comprised of bare ground, leaf litter and grass); (2) trunk; (3) branch; (4) log; and (5) other. We considered four explanatory variables: (1) substrate; (2) ground vegetation cover; (3) experimental CWD site; and (4) nest boxes. We also included social group and individual bird nested within social group as random effects. We included individual bird since we recorded numerous observations of each individual and social group because the Brown Treecreeper is gregarious and the behaviour of one individual may influence the behaviour of other group members. We then applied binomial GLMMs to investigate the relationship between target behaviours and the explanatory variables. Our statistical approach to contrast one category (in this case a category of behaviour) versus the rest of the categories (one-vs.-rest) is a legitimate approach to examine the effect of the experimental treatments on categories of the dependent variable according to our hypotheses. An alternative method would be to utilise a multinomial model that performs logistic regressions between categories of variables (e.g. category A vs. B, A vs. C, and A vs. D, when A is the baseline-category). However, our hypotheses examine how a particular behaviour, for example, is affected by the explanatory variables (substrate, ground vegetation cover, nest box treatment, coarse woody debris addition), rather than examining the difference between one behaviour versus a baseline behaviour. The multinomial model would also exaggerate the impact of variables due to the comparison with only the baseline-category instead of all the categories. Therefore, the current approach of repeated logit models is the most appropriate to examine our hypotheses.

We examined the significance of random factors for all analyses using a likelihood ratio test, which compared the deviances (2 times the log likelihood) of models with and without the random factor included [57], [58]. If removing the random factor caused a large enough drop in the log-likelihood, when compared to a chi-squared distribution with degrees of freedom equal to the number of additional models in the more complex model, then the factor was statistically significant. If the difference was not significant, we eliminated the random factor and GLMs were constructed.

To test our predictions that specific substrates should be used more frequently in the various experimental treatments for each of the individual behaviours, we constructed GLMMs and GLMs using a binomial distribution. Therefore, we analysed data from each behaviour type separately. For each model, we characterised the target substrate as 1 and all other substrates as 0. We conducted the analyses using the same random variables as in the previous analyses and using the fixed independent variables of (1) ground vegetation cover; (2) experimental CWD site; and (3) nest box.

For the logistic regression modelling, all possible models (full model vs. possible nested models) were considered using a backward elimination process to remove the least significant variables from the model using the Wald statistics. This was continued until all variables in the final model were statistically significant (P<0.05). We used this method since it is a standard statistical test for comparing nested models particularly when assessing fixed effects [58], [59], the experimental treatments were guided by the clear development of hypotheses, and the number of variables was small enough to consider all possible models (full model vs. possible nested models). Further, we were specifically interested in identifying only those individual variables that had significant effects, not on developing a best predictive model, so we deemed that variable selection (rather than model selection) was most suited to our needs. We conducted all statistical analyses using GenStat 13^th^ Edition.

## Results

We recorded a total of 1270 observations of behaviour and substrate use for 18 brown treecreeper individuals. We recorded observations for between two to 72 days for each bird, with an average of 43 (±6.01 s.e.) days. We recorded an average of 72.94 (±11.25 s.e.) observations per bird, with a range from three to 132. Large variations in the number of observations can be attributed to either an individual losing a transmitter early or the death of an individual. We observed brown treecreepers moving extensively through the reserves and across multiple polygons of varying treatment types [43]. Although some radio-tracked individuals did undertake separate dispersal movements, individuals were recorded within 10 m of other group members the majority of the time.

### Effect of Translocation

Our preliminary analyses found little difference in the behaviour and substrate use of brown treecreeper individuals when comparing observations taken pre- and post-settlement (Table 1). In particular, it could be expected that the level of observations on the ground would increase post-settlement due to newly released individuals displaying increased caution or avoiding substrates where they would be most vulnerable to predation [60]; however, there was no significant difference in the use of the ground pre- and post-settlement (P = 0.100). Although, there was a significantly increased use of trunks and decreased use of branches by the Brown Treecreeper post-settlement in comparison to pre-settlement (Table 1).

### Relationships between Behaviour and Substrate

**Figure 1:**
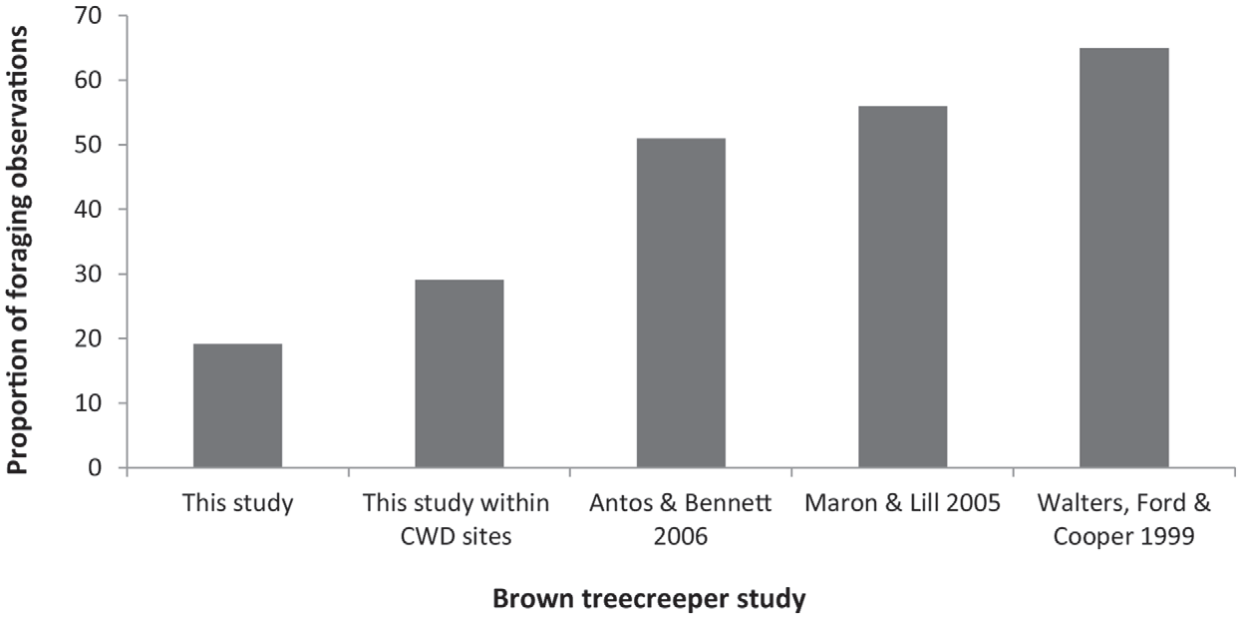
Brown treecreeper ground foraging observations in various studies. The proportion of foraging observations in which the bird was located on the ground, comparing results from this study, n = 1270; from this study within 1 ha experimental coarse woody debris (CWD) sites, n = 118; from Antos and Bennett [32], n = 644; from Maron & Lill [47], n = 126; and from Walters, Ford & Cooper [33], n = 1750.

Of 1270 observations on behaviour, 663 (52%) were of foraging, 374 (29%) were of vigilance and 155 (12%) were of resting and preening, with the remainder being calling (73, 6%) and other (5, 0.4%). We observed that vigilant and vulnerable behaviours occurred most frequently on trunks and logs. The majority of foraging behaviours occurred on trunks (58%), followed by ground substrates (19%). When individuals were within the 1 ha coarse woody debris sites, the proportion of foraging time on the ground increased to 30%, although this was lower than observed in previous studies on the brown treecreeper (Figure 1 ) [32], [33], [47]. For observations on the ground (10.6% of all observations), 70% occurred on leaf litter, followed by 19% on grass and 11% on bare ground.

### General Effects on Behaviour

The probability of a bird displaying a particular behaviour (foraging, vigilance or resting and preening) was not significantly influenced by ground vegetation cover, the addition of coarse woody debris, or the installation of nest boxes within the experimental polygon (Table 2). Neither did it vary between individuals or social groups. However, behaviour was significantly influenced by the substrate on which the individual was located (Table 2). Analysis of behavioural probabilities showed that the odds of a bird foraging when on the ground were greater than when on any other substrate, being 21.16 times the odds of a bird foraging on branches (Table 2). Further, the odds of a bird displaying vigilant and vulnerable behaviour were greatest when an individual was on the ‘other’ substrate category (e.g. stumps) followed by logs.

**Table 2:** Effects on brown treecreeper behaviour.

Behaviour	Factor	Estimate(± s.e.)	Odds ratio	?^2^	d.f.	P
**Foraging**	***Fixed Effects***					
	• Substrate			166.00	4	**<0.001**
	Ground	3.05 (**±**0.39)	21.16			
	Log	−0.93 (**±**0.20)	0.39			
	Other	−1.87 (**±**0.63)	0.15			
	Trunk	0.69 (**±**0.16)	1.99			
	• Vegetation			0.77	2	0.682
	• CWD site			0.18	1	0.667
	• Nest box			0.63	1	0.426
	• Constant	−0.20 (**±**0.19)				
	***Random effects***					
	• Group +	σ^2^ = 0.03				
	Group/Bird ID	σ^2^ = 0.00				0.423
**Vigilance**	***Fixed effects***					
	• Substrate			77.90	4	**<0.001**
	Ground	−3.06 (**±**0.57)				
	Log	0.63 (**±**0.19)				
	Other	1.10 (**±**0.40)				
	Trunk	−0.34 (**±**0.17)				
	• Vegetation			0.17	2	0.918
	• CWD site			1.29	1	0.256
	• Nest box			0.06	1	0.810
	• Constant	−0.70 (**±**0.22)				
	***Random effects***					
	• Group +	σ^2^ = 0.07				0.119
	Group/Bird ID	σ^2^ = 0.00				
**Resting and**	***Fixed effects***					
**Preening**	• Substrate			50.78	4	**<0.001**
	Ground	−3.27 (**±**0.93)	0.04			
	Log	0.44 (**±**0.24)	1.55			
	Other	0.47 (**±**0.47)	1.60			
	Trunk	−0.81 (**±**0.23)	0.45			
	• Vegetation			2.54	2	0.281
	• CWD site			2.05	1	0.153
	• Nest box			0.41	1	0.522
	• Constant	−1.75 (**±**0.25)				
	***Random effects***					
	• Group +	σ^2^ = 0.00				0.975
	Group/Bird ID	σ^2^ = 0.00				

Results of the generalized linear mixed models (GLMMs) and generalized linear models using binomial distribution (logit-link structure) which examined the influence of substrate and the three experimental treatments: (1) ground vegetation cover; (2) addition of coarse woody debris (CWD) in 1 ha sites; and (3) the installation of nest boxes; on the probability of an individual displaying three particular behaviours: (1) foraging; (2) vigilance; and (3) resting and preening. Group and individual bird nested within group were included as random effects in GLMMs (σ^2^ =  the variance of the random factor). Significant effects are shown in bold. Output shows the estimate and odds ratio for the significant substrate parameter in reference to the ‘branch’ category. Estimate for the constant is given from the full GLMMs. The total number of observations was 1270.

### Effects of Treatments on Behaviour on Particular Substrates

**Figure 2:**
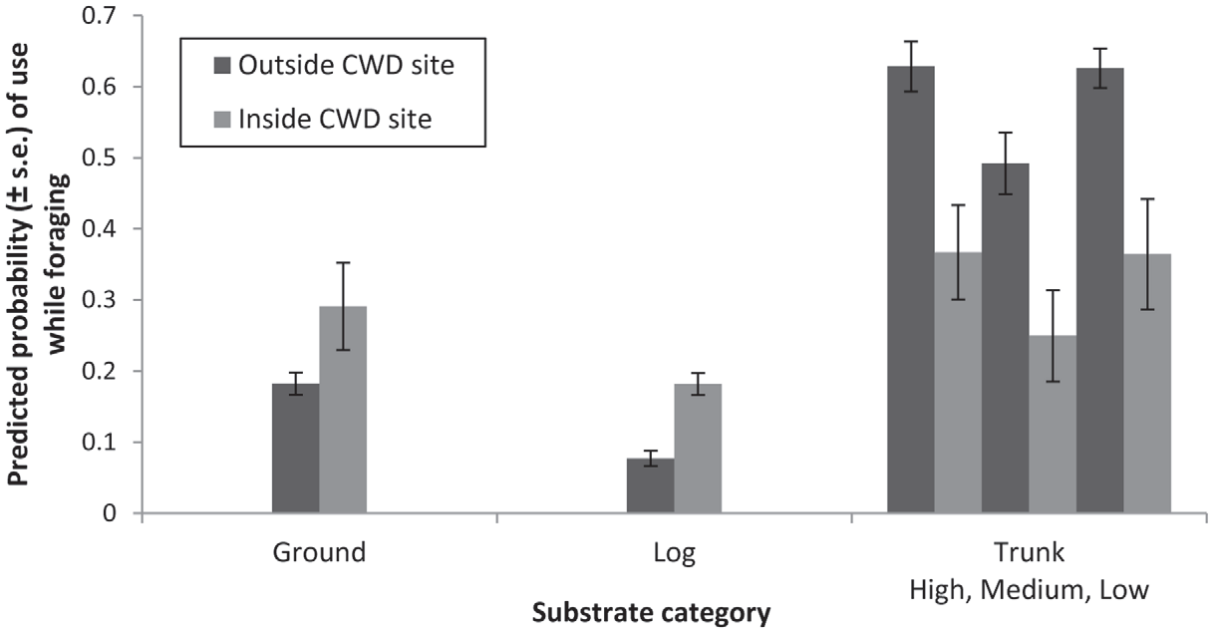
The effect of coarse woody debris site on substrate use by the brown treecreeper. The predicted probability (± s.e.) of a brown treecreeper using three target substrates whilst foraging. The use of these substrates was significantly influenced by whether an individual was within or outside an experimental coarse woody debris site (Ground: P = 0.053; Log: P = 0.010; Trunk: P<0.001). The use of trunks was also significantly influenced by the level of ground vegetation cover (high, medium, or low). Data presented were obtained by logit-link back-transformation.

When an individual was within a 1 ha coarse woody debris site there was an increased probability of foraging on a log or on the ground (Figure 2), and a decreased probability of foraging on a trunk (Table 3), compared with when an individual was outside the coarse woody debris sites. When examining data from individuals on ground-level substrates (on ground or on logs), there was a higher number of observations within two metres of a log (total of 81%; n = 313) than away from a log (19%; n = 72). Further, 61% of observations on the ground (n = 131) were within two metres of a log (either an experimental or a natural log). This suggests a preference by brown treecreepers to stay close to these structures since logs were sparse even within 1 ha coarse woody debris sites.

**Table 3:** Effects on brown treecreeper behaviour on particular substrates.

Target substrate	Behaviour	Parameter	Estimate (±s.e.)	Odds ratio	?^2^	d.f.	P
**Branch**	**Foraging**	***Fixed effects***					
		• Vegetation			19.72	2	**<0.001**
		Medium	0.61 (±0.27)	1.84			
		Low	−0.68 (±0.28)	0.51			
		• Nest box			3.94	1	**0.047**
		With boxes	−0.73 (±0.37)	0.48			
		• CWD site			0.82	1	0.365
		• Constant	−1.70 (**±**0.27)				
		***Random effects***					
		• Group +	σ^2^ = 0.14				0.080
		Group/BirdID	σ^2^ = 0.00				
**Branch**	**Vigilance**	***Fixed effects***					
		• Vegetation			11.67	2	**0.003**
		Medium	0.42 (±0.34)	1.52			
		Low	−0.71 (±0.33)	0.49			
		• CWD site			4.32	1	**0.038**
		In site	−1.17 (±0.56)	0.31			
		• Nest box			2.20	1	0.138
		• Constant	−0.97 (±0.28)				
		***Random effects***					
		• Group +	σ^2^ = 0.01				0.985
		Group/BirdID	σ^2^ = 0.00				
**Branch**	**Resting and**	***Fixed effects***					
	**Preening**	• Vegetation			6.20	2	**0.045**
		Medium	0.85 (±0.51)	2.34			
		Low	−0.30 (±0.47)	0.74			
		• CWD site			0.43	1	0.513
		• Nest box			0.35	1	0.553
		• Constant	−1.22 (±0.46)				
		***Random effects***					
		• Group +	σ^2^ = 0.00				0.985
		Group/BirdID	σ^2^ = 0.09				
**Ground**	**Foraging**	***Fixed effects***			1.11	2	0.574
		• Vegetation					
		• CWD site			3.75	1	0.053
		• Nest box			0.11	1	0.746
		• Constant	−1.60 (±0.23)				
		***Random effect***					
		• Group +	σ^2^ = 0.01				0.533
		Group/BirdID	σ^2^ = 0.07				
**Ground**	**Vigilance**	***Fixed effects***					
		• Vegetation			0.13	2	0.938
		• CWD site			0.02	1	0.883
		• Nest box			0.10	1	0.756
		• Constant	−4.06 (±1.01)				
		***Random effects***					
		• Group +	σ^2^ = 0.00				1.00
		Group/BirdID	σ^2^ = 0.00				
**Ground**	**Resting and**	***Fixed effects***					
	**Preening**	• Vegetation			0.05	2	0.975
		• CWD site			0.00	1	1.000
		• Nest box			0.00	1	1.000
		• Constant	−14.6 (±163)				
		***Random effects***					
		• Group +	σ^2^ = 0.00				1.000
		Group/BirdID	σ^2^ = 0.00				
**Log**	**Foraging**	***Fixed effects***					
		• CWD site			6.56	1	**0.010**
		In site	0.98 (±0.38)	2.65			
		• Vegetation			0.42	2	0.813
		• Nest box			0.17	1	0.678
		• Constant	−2.63 (±0.31)				
		***Random effects***					
		• Group +	σ^2^ = 0.03				0.842
		Group/BirdID	σ^2^ = 0.00				
**Log**	**Vigilance**	***Fixed effects***					
		• Vegetation			0.15	2	0.930
		• CWD site			1.52	1	0.218
		• Nest box			0.07	1	0.799
		• Constant	−0.84 (±0.29)				
		***Random effects***					
		• Group +	σ^2^ = 0.00				0.434
		Group/BirdID	σ^2^ = 0.11				
**Log**	**Resting and**	***Fixed effects***					
	**Preening**	• Vegetation			3.53	2	0.171
		• CWD site			0.65	1	0.421
		• Nest box			0.50	1	0.481
		• Constant	−0.38 (±0.45)				
		***Random effects***					
		• Group +	σ^2^ = 0.23				0.260
		• Group/BirdID	σ^2^ = 0.00				
**Trunk**	**Foraging**	***Fixed effects***					
		• Vegetation			7.99	2	**0.018**
		Medium	−0.56 (±0.23)	0.57			
		Low	−0.01 (±0.19)	0.99			
		• CWD site			11.68	1	**<0.001**
		In site	−1.07 (±0.31)	0.34			
		• Nest box			2.85	1	0.091
		• Constant	0.40 (±0.19)				
		***Random effects***					
		• Group +	σ^2^ = 0.00				0.083
		Group/BirdID	σ^2^ = 0.10				
**Trunk**	**Vigilance**	***Fixed effects***					
		• Vegetation			8.96	2	**0.011**
		Medium	−0.43 (±0.30)	0.65			
		Low	0.39 (±1.48)	1.48			
		• CWD site			0.87	1	0.351
		• Nest box			2.83	1	0.092
		• Constant	−0.51 (±0.27)		0.87	1	0.351
		***Random effects***					
		• Group +	σ^2^ = 0.00				0.647
		Group/BirdID	σ^2^ = 0.08				
**Trunk**	**Resting and**	***Fixed effects***					
	**Preening**	• Vegetation			1.64	2	0.441
		• CWD site			0.04	1	0.844
		• Nest box			0.29	1	0.590
		• Constant	−0.63 (±0.44)				
		***Random effects***					
		• Group +	σ^2^ = 0.15				0.393
		Group/BirdID	σ^2^ = 0.00				

Results of generalized linear mixed models (GLMMs) and generalized linear models (GLMs) using binomial distribution (logit-link structure) analysing the effects of (1) the level of ground vegetation cover (high, medium and low); (2) experimental coarse woody debris site (in or out of site); and (3) the presence or absence of nest boxes; on the probability of Brown Treecreeper individuals using each of the four target substrates: (1) branch, (2) ground, (3) log, and (4) trunk, whilst displaying the three types of behaviours (1) foraging, (2) vigilance, and (3) resting and preening. Group and individual bird nested within group were included as random effects in GLMMs (σ^2^ =  the variance of the random factor). The significant effects are shown in bold and include estimates (± s.e.) and odds ratios, which use high ground vegetation and outside of a 1 ha coarse woody debris site as the reference levels. Estimate for the constant is given from the full GLMMs. The total number of observations for each of the analyses by behaviour was: foraging: 663; vigilance: 374; and resting and preening: 155.

The level of ground vegetation cover did not significantly influence the use of the ground for any of the target behaviours (Table 3). We found that the presence of nest boxes significantly decreased the probability of foraging on branchs (χ^2^ = 3.94, d.f. = 1, P = 0.047), but did not significantly influence any other combinations of behaviour and substrate use. Similarly, there was no significant variation in substrate use from individual bird or social group.

## Discussion

We examined the effects of (or success of) experimental restoration treatments, specifically the addition of coarse woody debris, variations in ground vegetation cover, and installation of nest boxes, by quantifying the response of reintroduced brown treecreepers. To do this, we analysed the effect of these restoration treatments on the behaviour and substrate use of radio-tracked individuals. The key findings of our analyses were: (1) evidence of the benefits of the addition of coarse woody debris for foraging by the brown treecreeper; (2) little evidence of effects on behaviour and substrate use of variations in ground vegetation cover; and (3) limited use of ground substrates by individuals, with implications for restoration effectiveness.

### Addition of Coarse Woody Debris

Our data showed that individuals exhibited an increased probability of foraging when they were on a log or on the ground within the 1 ha coarse woody debris (CWD) sites. The proportion of ground foraging by the brown treecreeper when within these 1 ha sites, rather than when outside the 1 ha sites, is closer to the levels of ground foraging observed in previous studies (Figure 1) [32], [33], [47] and hence may improve the foraging efficiency of ground substrates. Further, when on the ground, individuals were often observed close to logs. Our results provide strong empirical confirmation of the benefit of coarse woody debris addition in our study area, and the success of this restoration treatment. This result was predicted at the outset of this investigation because the brown treecreeper is known to utilise coarse woody debris as a foraging substrate [32], [61].

We found a high level of use of logs, as well as trunks, for vigilant and vulnerable behaviours. However, there was no difference in the probability of these behaviours within versus outside CWD sites. The lack of an effect of CWD sites on bird behaviour did not support our predictions of CWD sites increasing the probability of vulnerable behaviours and decreasing vigilance. However, the high use of logs and trunks may have occurred because these substrates provide elevated locations from which individuals can gain a relatively unobstructed view to survey the surrounding environment for predators. Furthermore, an individual’s distance from a safe refuge is likely to influence their perceived predation risk [28], [29], [30], and these areas are likely to be close to important refuges in hollows in logs [25]. However, the coarse woody debris recently added to the reserves was generally not sufficiently decomposed to provide many hollows.

The benefits of coarse woody debris for the brown treecreeper may be improved through increasing timber loads in the reserves. Experimental redistribution of coarse woody debris has led to sustained increases in brown treecreeper numbers at loads of ≥40 tonnes/ha [62], [63], which is greater than the amount added to most coarse woody debris sites within Mulligans Flat and Goorooyarroo Nature Reserves [19].

### Installation of Nest Boxes

The installation of species-specific nest boxes did not significantly influence brown treecreeper behaviour. We did not have an appropriate opportunity to test the use of these structures specifically and did not observe any individuals utilising the nest boxes. However, there are many existing observations of the species using artificial hollows with a wide variety of characteristics [27]. It may be that the density of nest boxes was too low for individuals to reliably locate them, or for behaviour to be influenced by them. Alternatively, natural hollows may be abundant in the reserves, however we know that this is not the case based on comparisons of hollows in these reserves to other areas supporting the brown treecreeper [64]. Nest boxes were installed primarily to support breeding and roosting. Therefore, these structures may still be beneficial as our analyses examined only their secondary function of providing refuge from predators.

### Variation in Ground Cover

An unexpected finding from of our study was that ground vegetation cover did not significantly influence the behaviour and substrate use of reintroduced individuals. In particular, there was no significant effect of this treatment when individuals were on ground substrates. At the outset of this project, we predicted a higher use of the ground, particularly during foraging, in polygons with lower levels of ground vegetation cover, where invertebrates may be abundant and accessible [34], [35] and detection of and escape from predators easier [37]. The absence of a significant effect of ground vegetation cover on the use of ground substrates may have occurred because of the overall limited use of these substrates. Alternatively, calculation of ground vegetation cover at the polygon-level may be at a scale too large to detect any influence on behaviour and substrate use.

### Recovery of the Ground Layer

Brown treecreeper individuals spent 19% of their overall foraging time on the ground within the reintroduction site. This result contrasts with previous studies indicating that the species spends between 51–65% of its foraging time on the ground (Figure 1) [32], [33], [47]. Although the reintroduction process may alter a species’ ecology and hence influence the use of ground substrates [53], [65], [66], our preliminary analyses indicated that the use of the ground did not significantly differ between pre- and post-settlement. Therefore, it is unlikely that the reintroduction process greatly influenced the use of the ground.

Alternatively, an individual’s selection of foraging habitat may be affected by food abundance and accessibility [34], [35]. Mulligans Flat and Goorooyarroo Natures Reserves have been subject to a variety of degrading processes that may influence invertebrate abundance and consequently alter brown treecreeper foraging behaviour. These processes include livestock grazing [67], [68], and firewood harvesting which removes logs that provide habitat for invertebrates [69], [70]. This result highlights the importance of the management of the ground layer, particularly by promoting (1) the development of a cryptogamic crust [47], [71]; (2) an increased leaf litter layer, which is an important foraging substrate [32]; (3) reduced weed cover [47]; and (4) controlled levels of grazing pressure by exotic and native herbivores [23], [37]. It is possible that the existing restoration treatments, such as the addition of coarse woody debris and grazing management, may ultimately improve the ground layer, but there is a delay in realising their benefits. Similarly, these treatments may still be important and effective for the brown treecreeper even if their influence is not yet clear.

### Broader Implications

The results from this study highlight the unique information derived from the monitoring of behaviour and substrate use within an experimental framework. This study has three broad implications for ecological studies. First, through using the brown treecreeper as a bio-indicator, we were able to examine restoration success and identified the benefits that restoration manipulations can provide for fauna, specifically the addition of coarse woody debris. This demonstrates the value of examining the behaviour and substrate use of a focal species to understand the success and influence of restoration activities. Second, it is understood that successful reintroductions require comprehensive behavioural studies from existing populations. However, the limited use of ground substrates by reintroduced brown treecreeper individuals was unexpected given our existing knowledge on the behaviour of the species. This study indicates that behaviour and habitat use information from prior studies within a source population may not approximate that observed within a reintroduced population. Hence, there are potential difficulties in using existing research in other locations to inform habitat restoration and reintroductions. Last, our major findings emphasise the value of conducting species reintroductions within an experimental framework. They also highlight the value of linking restoration ecology with habitat ecology and behavioural ecology. This may be particularly the case for species reintroductions, which often produce highly unexpected outcomes.

## Supporting Information

Information S1 **Definitions of behaviour and substrate use recorded for reintroduced brown treecreepers.**
(DOC)
