# Supplementary material for: Habitat Selection and Behaviour of a Reintroduced Passerine: Linking Experimental Restoration, Behaviour and Habitat Ecology
Source: PLoS One. 2013 Jan 18;8(1):e54539. doi: 10.1371/journal.pone.0054539 (PMC3548787; doi:10.1371/journal.pone.0054539)
Supplement: Information S1 — Definitions of behaviour and substrate use recorded for reintroduced brown treecreepers. (DOC) [file pone.0054539.s001.doc]

**Information S1**

**Definitions of behaviour and substrate categories recorded for reintroduced Brown Treecreepers**

Substrates

The following are categories of substrate utilised by reintroduced Brown Treecreeper individuals throughout the monitoring period:

- Bare Ground – where the area within a 1 m diameter around an individual bird’s location was predominantly bare ground.
- Leaf litter – where the area within a 1 m diameter around an individual bird’s location was predominantly leaves.
- Grassy – where the area within a 1 m diameter around an individual bird’s location was predominantly grassy and the bird’s legs (at least) were obscured by the grass.
- Trunk – where the individual was perched on or moving along on a tree trunk, this includes dead trees.
- Branch – where the individual the bird was perched on or moving along the branches of a tree.
- Log – where the individual was perched on or moving along a log, where a log was defined as a section of a trunk or limb of a fallen tree fully or partially touching the ground that is greater than 10 cm in diameter along the majority of its length.
- <2 m of a log – where the individual was located within two metres of a log, this included if the individual was located on a log.
- Other – where the individual was on a substrate that does not correspond to the definition of the other categories listed above.

Behaviour

The following are categories of behaviour displayed by reintroduced Brown Treecreeper individuals throughout the monitoring period:

- Foraging – any type of activity that involved actively searching for or obtaining food items. This included the bird moving along a substrate and taking food from the surface, rapid movement along a substrate or extracting prey from beneath the surface of a substrate. This definition was chosen to maintain simplicity in recording behavioural observations and was based on previous knowledge of Brown Treecreeper foraging actions. The Brown Treecreeper relies mainly on gleaning as its foraging action , which can be defined as an individual moving along a substrate and removing prey from its surface .
- Resting – where an individual remained stationary and was not looking around in vigilance.
- Preening – all instances of body grooming or maintenance activities such as cleaning feathers or bill swiping where alternate sides of the bill are stroked against an object to clean the bill.
- Calling – where an individual was observed calling, generally in the form of contact calls or alarm calls.
- Vigilance – where the individual was stationary and was actively looking around scanning the area.
- Other – where the individual was observed displaying a behaviour that does not correspond to the definition of the other categories listed above.

**References**

1. Antos MJ, Bennett AF (2006) Foraging ecology of ground-feeding woodland birds in temperate woodlands of southern Australia. Emu 106: 29-40.

2. Maron M, Lill A (2005) The influence of livestock grazing and weed invasion on habitat use by birds in grassy woodland remnants. Biological Conservation 124: 439-450.
